# Design and validation of a 90K SNP genotyping assay for the water buffalo (*Bubalus bubalis*)

**DOI:** 10.1371/journal.pone.0185220

**Published:** 2017-10-05

**Authors:** Daniela Iamartino, Ezequiel L. Nicolazzi, Curtis P. Van Tassell, James M. Reecy, Eric R. Fritz-Waters, James E. Koltes, Stefano Biffani, Tad S. Sonstegard, Steven G. Schroeder, Paolo Ajmone-Marsan, Riccardo Negrini, Rolando Pasquariello, Paola Ramelli, Angelo Coletta, José F. Garcia, Ahmad Ali, Luigi Ramunno, Gianfranco Cosenza, Denise A. A. de Oliveira, Marcela G. Drummond, Eduardo Bastianetto, Alessandro Davassi, Ali Pirani, Fiona Brew, John L. Williams

**Affiliations:** 1 AIA-LGS Associazione Italiana Allevatori–Laboratorio Genetica e Servizi, Cremona, Italy; 2 Fondazione Parco Tecnologico Padano, Lodi, Italy; 3 Bioinformatics core, Parco Tecnologico Padano, Lodi, Italy; 4 Animal Genomics and Improvement Laboratory, BARC, USDA-ARS, Beltsville, Maryland, United States of America; 5 Department of Animal Science, Iowa State University, Ames, IA, United States of America; 6 Institute of Zootechnics, Università Cattolica del S. Cuore, Piacenza, Italy; 7 ANASB-Associazione Nazionale Allevatori Specie Bufalina, Centurano—Caserta, Italy; 8 Universidade Estadual Paulista (UNESP), Câmpus de Araçatuba, Sao Paulo, Brazil; 9 COMSATS Institute of Information Technology, Sahiwal, Pakistan; 10 Dipartimento di Scienze Zootecniche ed Ispezione degli Alimenti, Facoltà di Agraria, Università degli Studi di Napoli Federico II, Portici (NA), Italy; 11 Universidade Federal De Minas Gerais (UFMG), Belo Horizonte, Brazil; 12 Affymetrix UK Ltd, High Wycombe, United Kingdom; 13 Davies Research Centre, School of Animal and Veterinary Sciences, University of Adelaide, Roseworthy, SA, Australia; University of Florida, UNITED STATES

## Abstract

**Background:**

The availability of the bovine genome sequence and SNP panels has improved various genomic analyses, from exploring genetic diversity to aiding genetic selection. However, few of the SNP on the bovine chips are polymorphic in buffalo, therefore a panel of single nucleotide DNA markers exclusive for buffalo was necessary for molecular genetic analyses and to develop genomic selection approaches for water buffalo. The creation of a 90K SNP panel for river buffalo and testing in a genome wide association study for milk production is described here.

**Methods:**

The genomes of 73 buffaloes of 4 different breeds were sequenced and aligned against the bovine genome, which facilitated the identification of 22 million of sequence variants among the buffalo genomes. Based on frequencies of variants within and among buffalo breeds, and their distribution across the genome, inferred from the bovine genome sequence, 90,000 putative single nucleotide polymorphisms were selected to create an Axiom® Buffalo Genotyping Array 90K.

**Results:**

This 90K “SNP-Chip” was tested in several river buffalo populations and found to have ∼70% high quality and polymorphic SNPs. Of the 90K SNPs about 24K were also found to be polymorphic in swamp buffalo. The SNP chip was used to investigate the structure of buffalo populations, and could distinguish buffalo from different farms. A Genome Wide Association Study identified genomic regions on 5 chromosomes putatively involved in milk production.

**Conclusion:**

The 90K buffalo SNP chip described here is suitable for the analysis of the genomes of river buffalo breeds, and could be used for genetic diversity studies and potentially as a starting point for genome-assisted selection programmes. This SNP Chip could also be used to analyse swamp buffalo, but many loci are not informative and creation of a revised SNP set specific for swamp buffalo would be advised.

## Introduction

The water buffalo is a key species for smallholder producers in developing countries, and an important resource for specialized markets. Domestic buffaloes have a global distribution and are found in 129, mainly tropical and sub-tropical, countries. They contribute to the rural economies, especially in Asia, and in many regions where buffalo are more important than cattle. The world population of buffalo is about 195 million compared to more than 1.4 billion cattle, 1 billion sheep and 500–600 million goats [1]. There are two types of domestic water buffalo, the River Buffalo (*Bubalus bubalis bubalis*, 2n = 50) which has a global distribution, but are the predominant type found in the west from India to Europe. The second type, the Swamp Buffalo (*Bubalus bubalis carabanensis*, 2n = 48), is found more frequently in the eastern Asian countries, particularly from India through China, Indonesia and the Philippines. The buffalo population in Asia is more than 188 million, representing 95% of the world population [2–4]. In Africa domestic water buffalo are only found in Egypt, with about 4 million head, and more recently in Mozambique [4].

River type buffalo have been genetically selected for improved milk production: Mediterranean buffalo in Italy produce more than 2,000Kg of milk in a 270-day lactation with 8.1% fat and 4.6% protein, while in India the Jaffarabadi, and in Pakistan the Nili-Ravi, give more than 2,000Kg in a 319-day lactation with 7.6% fat [5]. Advanced reproductive technologies, including artificial insemination and embryo transfer, are routinely used where there is good buffalo husbandry to increase the rate of genetic gain. The swamp type buffalo, in contrast, has traditionally been found in extensive rural production systems providing traction in addition to a little milk and meat. Smaller than the river type buffalo, there has been little selection of swamp buffalo for production traits. Swamp buffalo typically produce 1-2Kg milk per day and average 350Kg milk per lactation [5].

Buffalo production systems vary widely in different countries depending on the local economy, climate, geography, cropping systems, size of the farms and primary purpose for buffalo production: milk, meat or draught. Buffaloes are kept in systems varying from extensive beef production, through rural multi-purpose systems to intensively managed herds for milk production [6, 7].

In Latin America, Murrah and Jaffarabadi river buffaloes were imported from India between the 1940s and 1960s [8, 9] to improve production from swamp buffalo which had been imported centuries before. More recently, Mediterranean buffalo have been imported from Italy. In Asia, native swamp type buffalo are being crossed or replaced by river type buffalo from India and Europe. The indiscriminate crossing of buffalo species and breeds results in a loss of local adaptation and the overall loss in genetic diversity of buffalo globally.

The use of molecular genetic approaches may increase the rate of gain in selection programmes and could be used to characterize, understand, and safeguard the global buffalo genetic diversity. The complete buffalo genome sequence and *de novo* assembly of an Italian Mediterranean river buffalo using MaSuRCA assembler [10] was completed by the International Water Buffalo Genome Consortium (GCF_000471725.1; deposited on NCBI in November 2013). A second genome sequence, of an Indian River buffalo is also available at NCBI with a read depth of 17–19X [11], which is aligned against the cattle assembly Btau 4.0 (accession PRJNA33659). The genome sequence is an important starting point for genetic and comparative genomic studies of buffalo. A SNP genotyping panel is required to further realise the potential of genomics for water buffalo genetics.

In this article, we describe the creation and testing of a genome-wide 90,000 SNP panel for the water buffalo. The performance of the panel was investigated in several buffalo populations and the panel was tested in a genome wide association study (GWAS) for milk production traits in Italian Mediterranean river buffalo.

## Results

### SNP sources

A total of 73 river buffaloes from 4 breeds (Italian Mediterranean, Murrah, Nili-Ravi, Jaffarabadi) were sequenced to a 5- to 12-fold depth using Illumina paired-end reads, yielding a total of 470X genome coverage. Buffalo sequences were aligned to the bovine UMD3.1 genome using Burrows-Wheeler Alignment tool (BWA) [12] and a total of 22,293,567 SNPs were discovered from the aligned sequences from the four river breeds.

### Axiom assay design

**Fig 1:**
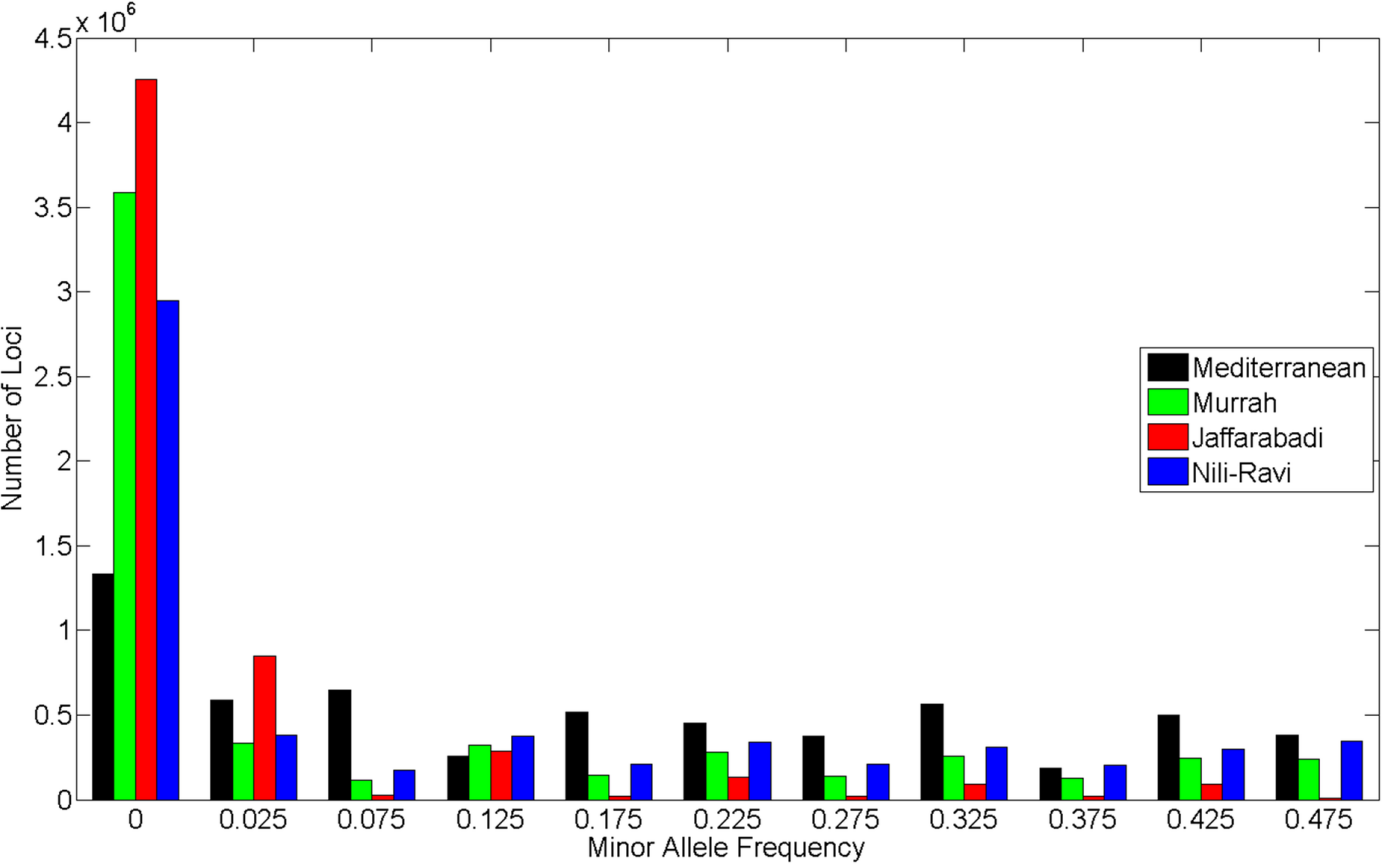
SNP MAF. Distribution of SNP Minor Allele Frequency (MAF) for Mediterranean, Murrah, Jaffarabadi and Nili-Ravi breeds used in the design of the SNP panel.

The distribution of SNPs on the buffalo genome was estimated by mapping the flanking sequence onto the UMD3.1 bovine genome sequence. The distribution of SNP minor allele frequencies (MAF) showed substantial differences among breeds (Fig 1), with the Jaffarabadi showing a markedly different frequency spectrum than the other 3 breeds having a large number of SNPs with low MAF.

The selection of candidate SNPs for the array design was based on genome-wide distribution with respect to the bovine UMD 3.1 genome, and was weighted with respect to significance of breed as follows: Mediterranean 30%; Murrah 30%; Jaffarabadi 20%; Nili-Ravi 20%. More than 16 million SNPs were selected for the development of the SNP assay, for which probesets were designed. The Affymetrix in-silico probeset design and evaluation pipeline predicts the performance of SNPs and calculates a conversion probability value using various criteria including: binding energy, GC content, and the expected degree of non-specific hybridization to multiple genomic regions. Regions that are highly repetitive in the genome, duplicated within the genome, contained an interfering SNP within 30bp from the candidate SNP or contained ambiguity were assigned a value of 0. The probesets with lowest predicted performance, with p-convert score of <0.56, were not considered. Approximately 9 million SNPs remained after applying these filters. A/T or G/C “allele-specific” SNPs were excluded as these SNPs require twice the number of probes on the Affymetrix chip compared with other SNP types. After all the filters had been applied 7.2 million SNPs remained which were then used to select the 90,000 markers.

For each SNP, where both probesets passed all filters, the probeset with the highest p-convert value was chosen giving 147,854 validated probesets of which 123,040 were selected for synthesis. An additional 1,179 markers were added from the BovineHD array based on cross reactivity and MAF in buffalo. The design also included 2000 non-polymorphic probes to assess sample quality during genotyping (DQC probes).

**Fig 2:**
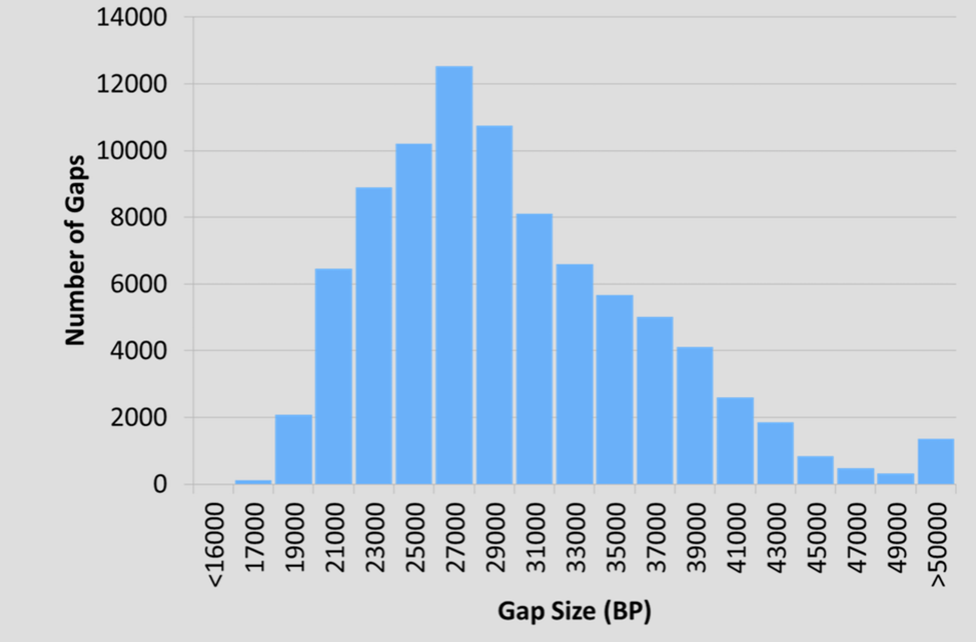
SNP GAP. The distribution of the gaps between the 90,000 SNPs with respect to their location in the bovine UMD 3.1 sequence.

The final Axiom® Buffalo Genotyping array design comprises 123,029 probes, which includes (single or double) probes to interrogate 89,988 SNPs, 5,799 QC probes and 1,784 gender determination probes. The distribution of the gaps between the 97,581 SNPs in relation to the bovine UMD 3.1 sequence was as follows: the minimum, maximum, 1-, 5-, 25-, 50-, 75–95-, and 99-percentile gap sizes were on average 17,462; 1,145,380; 19,566; 20,877; 24,718; 28,611; 34,085; 42,416; and 55,359 bp, respectively, across all loci (Fig 2).

### Axiom assay performance

The genome-wide Axiom® Buffalo Genotyping array was tested across 31 buffalo populations (15 River, 16 Swamp, 1 Cape buffalo, 1 Anoa) with a total of 1605 individuals (1376 River Buffalo, 200 Swamp Buffalo, 15 Cape Buffalo, 14 Anoa) to assess the performance of the chip. The data were processed through an automated open-source pipeline, AffyPipe [13], to extract and edit SNP probes [14]. A total of 67,330 and 9,229 SNP probes were validated as polymorphic and monomorphic high quality SNPs (PolyHighResolution and MonoHighResolution), respectively, considering all samples of river breeds. Fewer than 0.1% (83 SNPs) gave spurious signals with Variable Intensity Non-hybridizing Oligos (VINO cluster), which most likely arise from variations within the target probe sequence that were not identified in the sequence set used for the probe design. About 1.7% of the SNP (1,494) were missing one of the homozygous genotypes (NoMinorHom). Only 4.1% (3,668) of the probes had a call rate below the threshold. In total 9.1% of the SNP were rejected for low quality genotypes according to quality criteria used. Considering only the PolyHighResolution SNPs, the average sample call rate was 99.75% and the average sample reproducibility calculated by comparing the 26 replicate samples included in the sample set was 99.96%, demonstrating high quality and accuracy of the genotyping in the target river type buffalo.

The performance of the panel was also tested on more than 150 Swamp buffaloes from China, Thailand, Brazil and Indonesia. Genotyping success for the Swamp buffalo was, as may be expected, lower than for the river buffalo: 23,938 SNPs (24.5%) were polymorphic high resolution, while 47,016 (48.2%) were high quality monomorphic SNPs. In total 26,627 (27.3%) of the SNPs were rejected as low quality, i.e. where call rates were below the threshold for calling, missing a homozygote or genotypes were poorly separated which is possibly caused by polymorphisms in probe target sequences. The distribution of polymorphic SNPs in River and Swamp buffalo across the genome is shown in Table 1. The panel was also tested in Anoa (*Bubalus depressicornis*) and in the distantly related wild Cape buffalo. Results for 14 samples from Anoa and 15 from Cape buffalo (*Syncerus caffer*) from South Africa indicated that 7,652 (7.8%) and 3,239 (3.3%) of the SNPs were polymorphic high resolution in these species respectively suggesting these are ancestral variations, while 52,425 (53.7%) and 65,641 (67.3%) were high quality monomorphic SNPs.

**Table 1:** Numbers of heterozygous SNP per chromosome. SNPs are annotated per bovine chromosome as the buffalo genome is not yet assigned to chromosomes.

Buffalo BTA (BBC)	Number of heterozygous SNPs in river buffalo	Number of heterozygous SNPs in swamp buffalo
1	4048	2768
2	3525	2409
3	3135	2058
4	3068	1909
5	3022	1942
6	2982	1765
7	2890	1795
8	2828	2077
9	2620	1594
10	2656	1620
11	2762	1933
12	2172	1413
13	2173	1419
14	2233	1416
15	2084	1474
16	2062	1278
17	1884	1317
18	1668	998
19	1639	970
20	1813	1335
21	1789	1240
22	1609	1064
23	1346	900
24	1691	954
25	1202	817
26	1347	879
27	1191	866
28	1223	714
29	1249	796

### Application to genome-wide association study (GWAS)

A GWAS was performed using 529 individuals for which milk records were available from 4 Italian Mediterranean buffalo farms. These were genotyped with the 90K SNP panel giving 66,534 high-quality (PolyHighRes) SNPs with MAF >1%. As the draft genomic sequence of the buffalo (GCF_000471725.1) is currently not assigned to chromosomes, chromosome and position for all SNPs were based on the bovine UMD 3.1 genome sequence. This also facilitated the use of the bovine gene annotation information.

**Fig 3:**
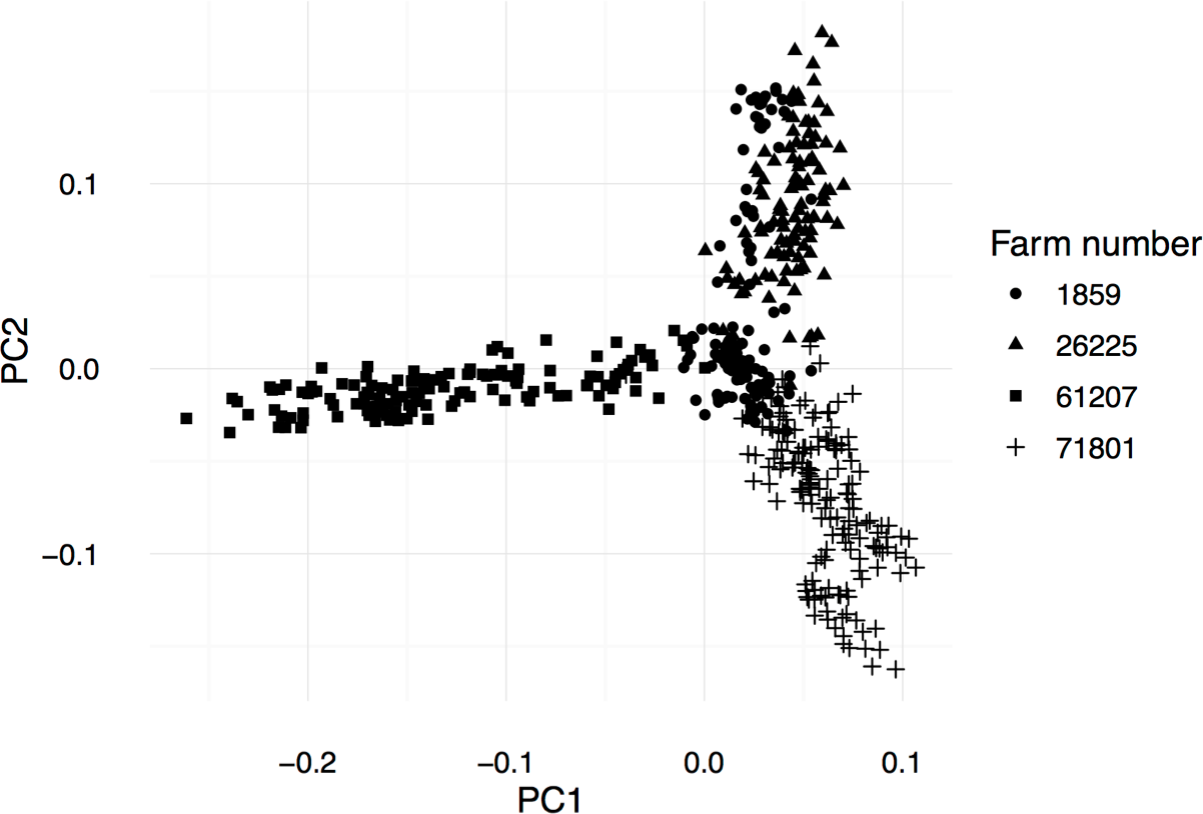
MDS. Multidimensional scaling plot showing the relationship among individuals from the four farms used in this study.

Analysis of the population structure from the genotype data showed a strong farm of origin effect. One main cluster included animals from 2 farms (*1859* and *26225)*, while individuals from the other two farms (*71801* and *61207)* clustered separately, showing only minimal overlap with the other farms (Fig 3). This is explained by the use of same artificial insemination (AI) bulls by farms *1859* and *26225*, hence the observed genetic overlap. Farms *71801* and *61207* used mainly natural service bulls, which were less unrelated.

**Fig 4:**
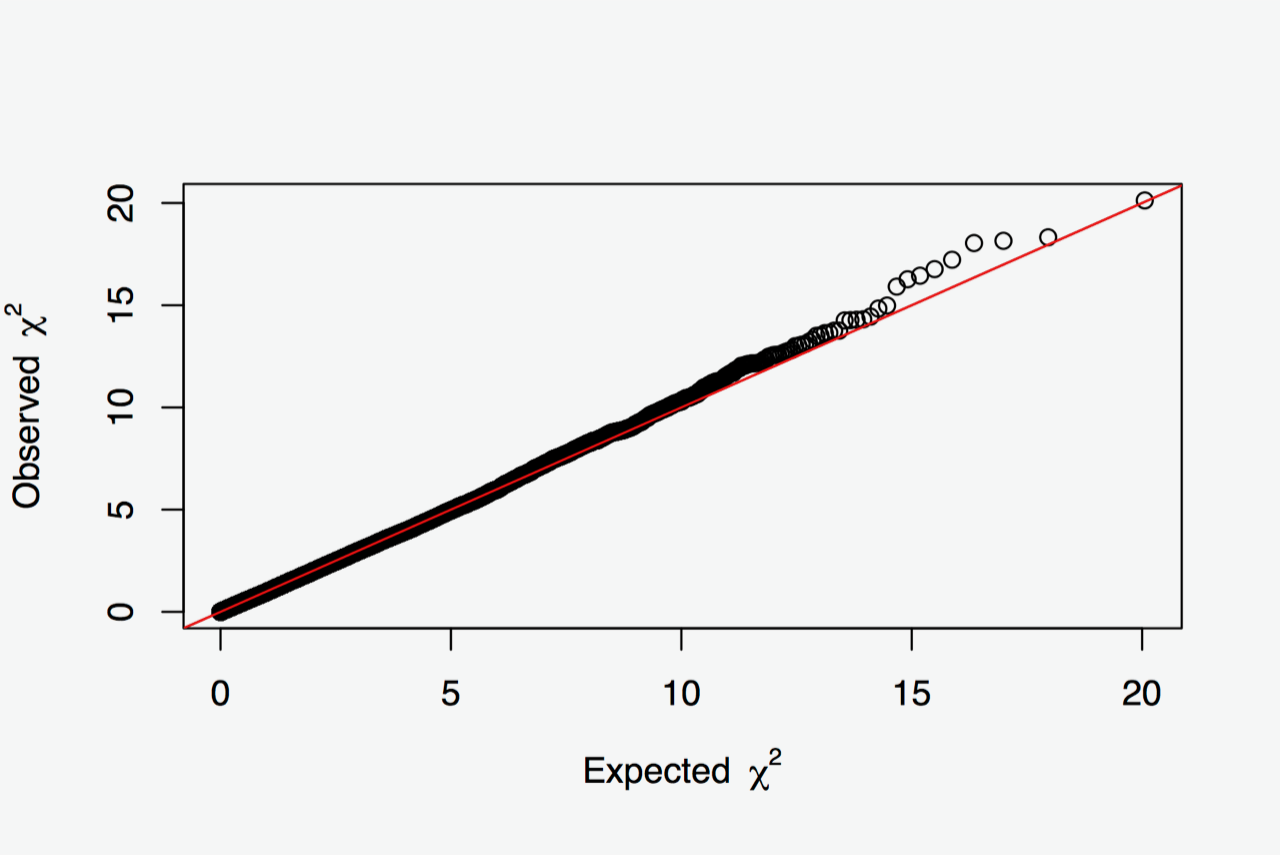
Q-Q plot. Quantile-Quantile plot showing P-values from GWAS. Deviations from the distribution under the hypothesis (null hypothesis of no association) are showed.

A genome-wide association analysis was performed with the GenABEL R package, using the GRAMMAR procedure [15]. First, an additive polygenic model was fitted to obtain individual residuals using the genomic relationship matrix. Then, the SNP association was tested using a linear model on residuals from the first step. The SNP statistical significance was corrected by the stratification of the population using the Genomic Control option [16] (Fig 4). Milk yield heritability, based on a classical animal model and recorded pedigree which was used to build an additive relationship matrix, was 0.38, whereas it increased to 0.45 using the genomic relationship matrix. These values are similar to those obtained in cattle for the same trait [17].

**Fig 5:**
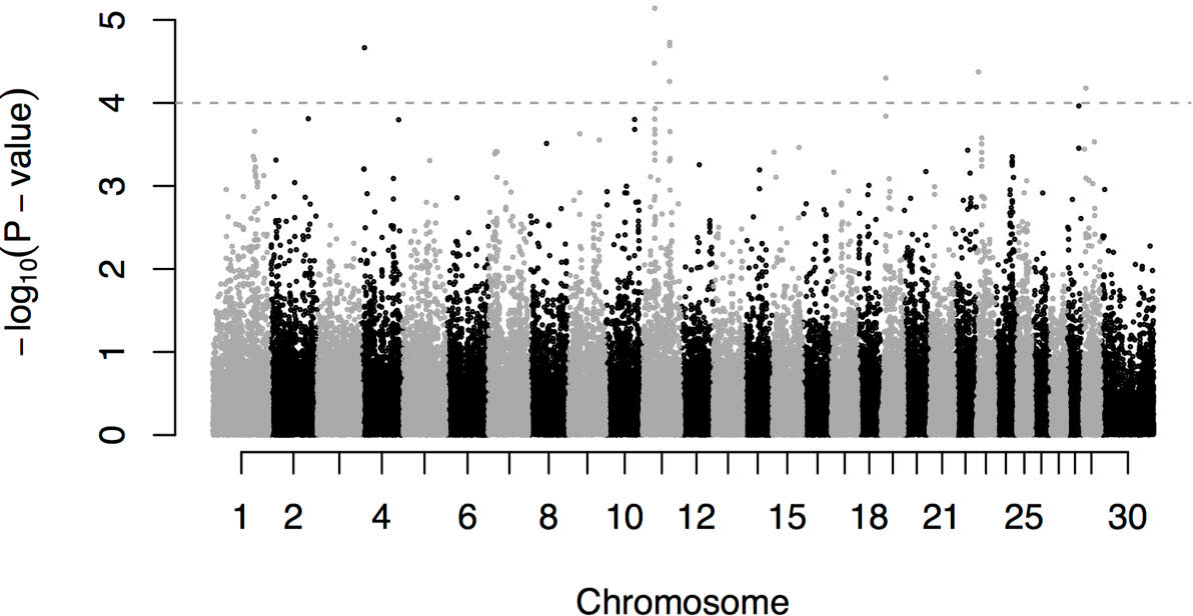
Association between individual SNP and milk yield from a genome-wide association study. Each dot represents a SNP that has passed the quality threshold and the high above the X axis is proportional to the strength of the association.

A total of nine significant associations were found on the bovine-based chromosomes (BBC) 4, 11 19, 23 and 29 (Fig 5). Among these, five SNPs (including the SNP with the lowest P-value) were identified in two regions of BBC11 at ~33Mb and ~76Mb (Table 2). The most significant association (P-value < 7.24 x 10–06) was for two SNPs in the 33Mb region of BBC11 (AX-85080229 and AX-85093842). The significant SNP on BBC4 (AX-85143079) was located (~51 Kb) downstream of Collagen alpha-2 gene (*COL1A2*) which is associated with the kinetics of milk production in sheep [18].

**Table 2:** Significant SNPs obtained in the GWA study for milk yield in 529 Italian Mediterranean buffalo.

SNP	BTA	Position	N	*P*-val
AX-85080229	11	34107047	529	7.24E-06
AX-85114201	11	76258496	529	1.86E-05
AX-85125077	11	75854702	529	2.04E-05
AX-85143079	4	11676240	529	2.16E-05
AX-85093842	11	32955361	529	3.32E-05
AX-85048470	23	6051649	529	4.23E-05
AX-85140457	19	13338605	528	5.01E-05
AX-85041172	11	75787638	528	5.49E-05
AX-85086756	29	9205713	528	6.64E-05

## Discussion

The present work describes the design and testing of a genome-wide SNP panel for water buffalo. Over 5.8 million high quality SNPs were discovered in 4 breeds of river buffalo, 3 from the Indian sub-continent and the 4^th^ from Italy. The distribution of SNP minor allele frequencies (MAF) differed among breeds (Fig 1). These data were used to design a SNP assay for genomic studies in river buffalo. An Affymetrix genotyping array with 123,040 probes was created to interrogate 90,000 SNPs. This array was tested across River and Swamp buffalo, Anoa and the wild African Cape buffalo. The panel had 66,534 polymorphic high quality SNPs with a MAF greater than 1% when tested in Italian river buffalo, and a further 10,025 high quality SNP loci which were not polymorphic. These later loci may prove to be polymorphic in other populations. The Axiom array was also tested in Swamp buffalo, which is an evolutionarily distinct species with 48 chromosomes compared with 50 of the river buffalo, resulting from a fusion of river buffalo chromosomes 4 and 9 in swamp buffalo [19]. Estimates based on molecular data (mitochondrial DNA, microsatellite loci and biochemical markers) suggest the species diverged 150,000 to 1.7 million years ago [20–22]. In total 23,938 SNPs were polymorphic in Swamp buffalo with a MAF of 1% or greater, which may be ancestral variation that existed prior to species divergence. A further 47,016 loci were of good quality, but non-polymorphic in the samples used for the panel validation. These loci show sequence conservation at probe binding sites, but it is likely that the polymorphism occurred in River buffalo after species divergence. Among the 97,581 SNPs on the Axiom array, 3,239 were also polymorphic in the wild African Cape Buffalo, which is estimated to have diverged from the river buffalo in the Miocene period 5–23 million years ago [23]. These SNPs therefore are likely to represent historic variations originating from a common ancestor prior to divergence of domestic and wild buffalo species.

Pedigree information is essential to calculate estimated breeding values (EBV), but is also needed to measure and control inbreeding. Even though, pedigree information is often unavailable for buffalo populations, the use of SNP information can be used to reconstruct pedigrees and resolve relationships among individuals to support breeding programs. Genomic relationship can be more accurate than traditional pedigrees which routinely contain errors [24, 25]. In the present study the heritability of milk traits increased from 0.38 calculated using recorded pedigrees to 0.45 using the genomic relationship. Inbreeding can also be estimated from SNP data, e.g. using Runs of Homozygosity [26]. Controlling inbreeding is important to maintain genetic diversity and to avoid the expression of recessive defects. This is especially important in small local populations where buffaloes are bred by smallholders with few animals and often using a common bull.

The SNP data can be used to disentangle the genetic structure of buffalo populations at the farm level, as demonstrated here using the 90K SNP array where we show that farm of origin can be distinguished, when stock bulls are used. Conversely the convergence of genetic diversity among farms is observed where a small number of AI bulls is used, as is the case of the Mediterranean buffalo.

For swamp buffalo, nearly 24K SNPs on the Axiom array were polymorphic with a MAF greater than 1%. A large number of SNP loci in river buffalo were monomorphic in the swamp buffalo, suggesting that these polymorphisms occurred following the divergence of the two buffalo species. Nevertheless, there are sufficient polymorphic loci on the panel e.g. to explore the diversity of the swamp buffalo. Swamp buffalo are being upgraded for milk production by crossing with the more productive river buffalo in countries such as China, the Philippines and South America [27]. The SNP panel can be used to study introgression of the different species and estimate the level of admixture.

**Fig 6:**
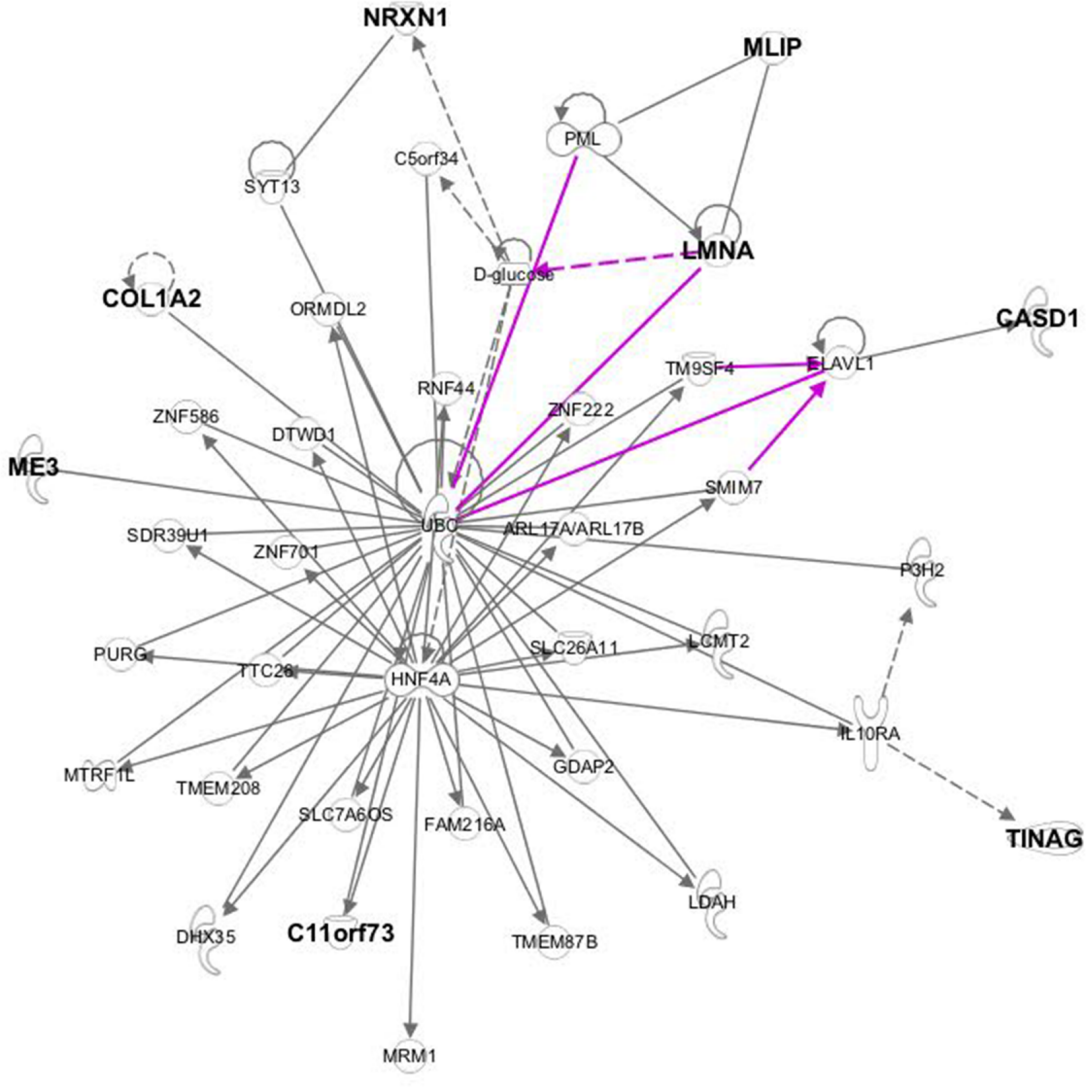
IPA merged network. The focus molecules are indicated in bold. One of the link molecules in the merging networks is D-Glucose, important for the milk synthesis and involved in the gluconeogenesis pathway.

**Fig 7:**
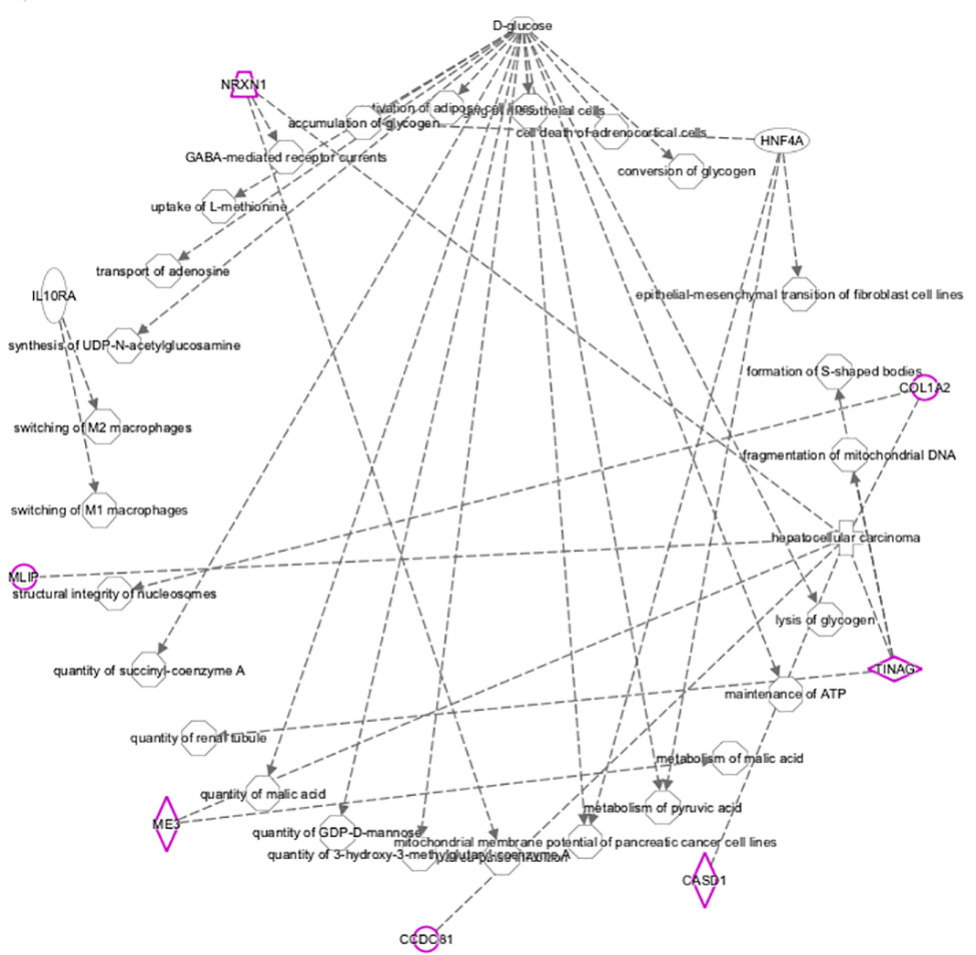
IPA molecule function network. The focus molecules are highlighted in purple. This network shows the central role of D-Glucose molecule and its connections with the function of the molecules close to significant SNPs in the present study.

The SNP array reported here was used to perform a genome-wide association study which identified nine significant SNPs associated with milk production. These SNPs were mapped onto bovine chromosomes 4, 11, 19, 23 and 29. Using the annotation of the bovine genome (UMD 3.1) putative candidate genes associated with milk yield close to significant SNPs were identified and retrieved using Ensembl BIOMART. The networks within which these genes mapped are related to cell morphology, cellular function and maintenance, cell cycle and organ morphology. The merged network, which includes most of the genes close to significant SNPs (Fig 6), shows enrichment in the metabolic pathways, particularly Gluconeogenesis I (p-value 9,71E^-03^). The significant SNP on BBC4 (AX-85143079) is located ~51 Kb downstream of *COL1A2* gene, which has been associated with the kinetics of milk production in sheep, with an under expression of *COL1A2* found in low-milk flow ewes [18]. SNPs in the intronic region of *COL1A2* have been associated with milk fat yield in cattle [28]. *COL1A2* is a focus gene for the functional network for glucose synthesis and may link together genes near to other significant SNPs from the GWAS analysis (Fig 7). *COL1A2* is therefore a good candidate gene for milk yield in buffalo while the supply of glucose to the mammary gland is critical for milk production and hence this is a relevant network for further study.

Other genes near to the significant SNPs are not obvious candidates for milk production traits. The two most significant SNPs, located on BBC 11 (AX-85080229 and AX-85093842), are 120Kbp downstream of Neurexin-1 (*NRXN1*), which is involved in cell signalling. The closest gene to the significant SNP on BBC23 (AX-85048470) is tubulointerstitial nephritis antigen (*TINAG*), a gene involved in immune response. The other two significant SNPs, AX-85140457 on BBC19 and AX-85086756 on BBC29 are close to genes involved in mitochondrial RNA methylation (Mitochondrial rRNA Methyltransferase 1 Homolog–*MRM1*) and in the cell structure (Coiled-Coil Domain Containing 81- *CCDC81*), respectively.

Genetic breeding programs for livestock are increasing using high-density genotyping panels which are comprised of SNPs selected to evenly span the genome [29, 30]. However, inclusion of putative causal mutations with large effect, such as those identified from GWAS, within genotyping panels are used in estimating genomic breeding values and have been shown to increase the accuracy [31]. Therefore refinement of this panel may take into account GWAS data such as that presented here and should also consider the inclusion of addition loci selected to be polymorphic in swamp buffalo.

The use of genetically improved river buffalo breeds will ensure milk supply for poor communities, but using semen from a limited number of improved bulls will threaten the genetic diversity of local populations. This is particularly the case for swamp buffalo which are being replaced or crossed with the more productive river buffalo. The SNP array described here, if used appropriately, will facilitate the identification of genes of major effect or the application of genomic selection to enhance the genetic improvement of buffalo, while being used to monitor and manage cross breeding programmes to maintain genetic diversity at a global level.

## Materials and methods

### Animals and DNA samples

DNA sequences for SNP discovery came from Pakistan—Nili-Ravi breed, Italy—Mediterranea italiana breed, and Brazil—Murrah and Jaffarabadi breeds. Samples genotyped to test the SNP array were provided by members of the International Water Buffalo Genome Consortium including: China, Indonesia, Thailand, Pakistan, Philippines, Iran, Egypt, Mozambique, Romania, Italy, Colombia and Brazil. Cape Buffalo were provided by Stellenbosch University (South-Africa) and Anoa by Antwerp Zoo (Belgium) and Glamorgan University (UK).

Samples for the GWAS came from four commercial farms in Italy and all animals sampled belonged to the Mediterranea italiana breed. Buffy coat was obtained by centrifuging fresh blood at 4000G for 20 minutes at 4°C followed by 2 washes for 10 minutes first with sterile water then 0.9% NaCl. Buffy coat samples were resuspended in the lysis buffer [Tris-EDTA (100mM Tris-HCL pH 8.0, 10mM EDTA pH 8.0), 250mM NaCl] and preserved at -20°C until DNA extraction. DNA was extracted from thawed buffy coat using a classic procedure by incubating at 55°C per 30 minutes with proteinase K (20mg/mL, Sigma-Aldrich, USA) and 10% SDS followed by 2 phenol-chloroform (1:1, 1V) extractions and a chloroform extraction [32]. DNA was precipitated by adding 2 volumes 100% ethanol, the pellet washed with 70% ethanol, dried and then dissolved in 1X TE (100mM Tris-HCL pH 8.0, 10mM EDTA pH 8.0). All the samples were collected during routine veterinary checks according to ethical rules in the countries participating to the International Water Buffalo Genome Consortium (IWBGC).

### Construction of libraries and sequencing

The IWBGC produced sequence for 86 buffaloes from 8 breeds with a depth between 5 and 12X by Illumina paired-end reads, yielding a total of 470X genome coverage. Of these data, sequences of 4 breeds were used for SNP discovery yielding a total of 22,293,567 SNPs.

### SNP discovery

Buffalo sequences were aligned to the bovine UMD3.1 genome using BWA software version 0.5.9 (http://bio-bwa.sourceforge.net/) [20]. Aligned sequences were processed with SAMtools version 0.1.18 [33] and Picard tools [34] in order to format the data for SNP calling with the UnifiedGenotyper of the genome analysis tool kit GATK [35]. All computation steps related to sequence alignment and SNP calling was conducted using the iAnimal cyberinfrastructure system [36].

Only SNPs that were heterozygous in at least one individual within each buffalo breed were kept for. The heterozygous SNPs were filtered and only those that had a base pair quality score of Q>10 and did not have another SNP within 10bp were retained, to maximize genotyping probe efficacy. The final unique, filtered SNP list was used for genotyping all sequenced individuals. These SNPs were genotyped from the original unfiltered SNP data for each individual (e.g. a filtered VCF file including heterozygous and homozygous SNPs, regardless of the quality score). These steps were repeated for each breed and for the combined data (concatenation of all breeds). The allele frequencies for these SNPs were calculated in each breed as well as across the breeds and the minor allele frequencies (MAF) were then estimated.

The FastaAlternateReference function from the Genome Analysis Toolkit was used to create a “buffalo corrected” version of the Bos taurus UMD3.1 reference. The corrected reference was identical to the bovine genome, except for targeted changes to loci where homozygous SNPs were identified across all buffalo individuals when compared to the bovine genome to reflect the true buffalo sequence. Flanking sequence was retrieved from the buffalo corrected reference to create probes for all filtered SNPs.

### SNP selection for the assay design

The algorithm used to select SNP for the assay was based on that used to select SNP for the Illumina BovineSNP50 [37]. This approach emphasizes quality of SNP sources by using “waves” of SNP of descending quality. The definitions of the waves used in this chip design are included in Table 3. For SNP i and breed j, a score was calculated as

$${score}_{i}=\sum_{j}^{groups}\left(w_{j}\times {MAF}_{i,j}\times [B_{i,j}-A_{i,j}]\times (1-\frac{\left|\left(A_{i,j}+B_{i,j}\right)/2-P_{i}\right|}{\left[B_{i,j}-A_{i,j}\right]/2})\right)$$

where w_j_ is the weight used for each breed, MAF_i,j_ is the minor allele frequency of locus i in breed j, A_i,j_ and B_i,j_ are the starting and ending positions of the current gap for breed j that contains locus i, and P_i_ is the location of SNP_i_. At each round of SNP selection, the SNP with the highest score is selected for addition to the assay, the endpoints (A_i,j_ and B_i,j_) are updated for SNP where P_i_ is contained in the existing gap, i.e. P_i_ ϵ [A_i_,B_i_]. It should be noted that the endpoints of the gap surrounding P_i_ are the nearest flanking loci on each side that are polymorphic in breed j.

**Table 3:** Criteria of SNP used in each wave of SNP selection, total number that met the criteria, and number used from that wave.

Wave	Total SNP	SNP Used	Nearest SNP	Oligos^1^	Design Score^2^	SNP Score^3^	MAF^4^			
							Medit	Murr	Jaff	NR
1	114	113	>30 bp	1	0.8	100	>0.20	>0.20	>0.20	>0.20
2	379	371	>30 bp	1	0.8	100	>0.10	>0.10	>0.10	>0.10
3	72	72	>30 bp	1	0.8	100	>0.05	>0.05	>0.05	>0.05
4	111	108	>30 bp	2	0.8	100	>0.05	>0.05	>0.05	>0.05
5	5,022	4,042	>30 bp	1	0.7	100	>0.20	>0.20	>0.20	>0.20
6	14,862	8,452	>30 bp	1	0.7	100	>0.10	>0.10	>0.10	>0.10
7	3,087	1,399	>30 bp	1	0.7	100	>0.05	>0.05	>0.05	>0.05
8	3,349	1,304	>30 bp	2	0.7	100	>0.05	>0.05	>0.05	>0.05
9	4,201	1,721	>30 bp	1	0.7	50	>0.20	>0.20	>0.20	>0.20
10	11,042	3,818	>30 bp	1	0.7	50	>0.10	>0.10	>0.10	>0.10
11	3,157	1,004	>30 bp	1	0.7	50	>0.05	>0.05	>0.05	>0.05
12	2,641	786	>30 bp	2	0.7	50	>0.05	>0.05	>0.05	>0.05
13	4,348	1,155	>30 bp	1	0.6	100	>0.20	>0.20	>0.20	>0.20
14	11,621	2,428	>30 bp	1	0.6	100	>0.10	>0.10	>0.10	>0.10
15	2,461	553	>30 bp	1	0.6	100	>0.05	>0.05	>0.05	>0.05
16	3,243	641	>30 bp	2	0.6	100	>0.05	>0.05	>0.05	>0.05
17	4,011	1,022	>30 bp	1	0.6	50	>0.20	>0.20	>0.20	>0.20
18	9,293	1,936	>30 bp	1	0.6	50	>0.10	>0.10	>0.10	>0.10
19	2,797	581	>30 bp	1	0.6	50	>0.05	>0.05	>0.05	>0.05
20	2,842	540	>30 bp	2	0.6	50	>0.05	>0.05	>0.05	>0.05
21	925	134	>30 bp	1	0.8	100	>0	>0	>0	>0
22	38,340	4,551	>30 bp	1	0.7	100	>0	>0	>0	>0
23	28,103	2,896	>30 bp	1	0.7	50	>0	>0	>0	>0
24	27,644	2,039	>30 bp	1	0.6	100	>0	>0	>0	>0
25	22,351	1,876	>30 bp	1	0.6	50	>0	>0	>0	>0
26	186	7	>30 bp	2	0.8	100	>0	>0	>0	>0
27	5,550	299	>30 bp	2	0.7	100	>0	>0	>0	>0
28	4,186	295	>30 bp	2	0.7	50	>0	>0	>0	>0
29	5,311	308	>30 bp	2	0.6	100	>0	>0	>0	>0
30	4,123	312	>30 bp	2	0.6	50	>0	>0	>0	>0
31	11	1	>10 bp	1	0.8	100	>0.20	>0.20	>0.20	>0.20
32	36	0	>10 bp	1	0.8	100	>0.10	>0.10	>0.10	>0.10
33	7	0	>10 bp	1	0.8	100	>0.05	>0.05	>0.05	>0.05
34	5	0	>10 bp	2	0.8	100	>0.05	>0.05	>0.05	>0.05
35	436	37	>10 bp	1	0.7	100	>0.20	>0.20	>0.20	>0.20
36	1,030	65	>10 bp	1	0.7	100	>0.10	>0.10	>0.10	>0.10
37	191	11	>10 bp	1	0.7	100	>0.05	>0.05	>0.05	>0.05
38	245	19	>10 bp	2	0.7	100	>0.05	>0.05	>0.05	>0.05
39	453	48	>10 bp	1	0.7	50	>0.20	>0.20	>0.20	>0.20
40	825	61	>10 bp	1	0.7	50	>0.10	>0.10	>0.10	>0.10
41	282	20	>10 bp	1	0.7	50	>0.05	>0.05	>0.05	>0.05
42	228	18	>10 bp	2	0.7	50	>0.05	>0.05	>0.05	>0.05
43	584	68	>10 bp	1	0.6	100	>0.20	>0.20	>0.20	>0.20
44	1,045	55	>10 bp	1	0.6	100	>0.10	>0.10	>0.10	>0.10
45	226	18	>10 bp	1	0.6	100	>0.05	>0.05	>0.05	>0.05
46	356	29	>10 bp	2	0.6	100	>0.05	>0.05	>0.05	>0.05
47	673	84	>10 bp	1	0.6	50	>0.20	>0.20	>0.20	>0.20
48	999	104	>10 bp	1	0.6	50	>0.10	>0.10	>0.10	>0.10
49	379	38	>10 bp	1	0.6	50	>0.05	>0.05	>0.05	>0.05
50	344	41	>10 bp	2	0.6	50	>0.05	>0.05	>0.05	>0.05
51	61	6	>10 bp	1	0.8	100	>0	>0	>0	>0
52	2,067	76	>10 bp	1	0.7	100	>0	>0	>0	>0
53	1,701	78	>10 bp	1	0.7	50	>0	>0	>0	>0
54	1,956	98	>10 bp	1	0.6	100	>0	>0	>0	>0
55	1,805	121	>10 bp	1	0.6	50	>0	>0	>0	>0
56	11	0	>10 bp	2	0.8	100	>0	>0	>0	>0
57	325	13	>10 bp	2	0.7	100	>0	>0	>0	>0
58	299	14	>10 bp	2	0.7	50	>0	>0	>0	>0
59	384	17	>10 bp	2	0.6	100	>0	>0	>0	>0
60	371	28	>10 bp	2	0.6	50	>0	>0	>0	>0
61	662,323	28,696	>30 bp	1	0.6	50	>0 for 3 breeds			
62	105,202	1,384	>30 bp	2	0.6	50	>0 for 3 breeds			
63	41,051	441	>10 bp	1	0.6	50	>0 for 3 breeds			
64	7,236	62	>10 bp	2	0.6	50	>0 for 3 breeds			
65	988,679	10,457	>30 bp	1	0.6	50	>0 for 2 breeds			
66	154,274	728	>30 bp	2	0.6	50	>0 for 2 breeds			
67	63,443	251	>10 bp	1	0.6	50	>0 for 2 breeds			
68	10,525	44	>10 bp	2	0.6	50	>0 for 2 breeds			
69	3,022,893	0		1						
70	502,138	0		2						
Total	5,800,471	87,894								

^1^Number of oligonucleotide probes needed to assay the SNP.

^2^Design score generated by Affymetrix

^3^SNP score indicating likelihood of a real SNP at the location generated by GATK.

^4^Minor allele frequency for Mediterranean (Medit), Murrah (Murr), Jaffarabadi (Jaff) and Nili-Ravi (NR).

### Affymetrix design pipeline

The probes on the Axiom Buffalo Genotyping Array 90K were designed using the standard Affymetrix design pipeline. At the time of writing, more than a hundred custom Axiom arrays have been designed using this pipeline.

The Affymetrix design pipeline uses a number of stringent rules to identify the SNPs with the highest probability of conversion. At the end of this process, all the SNPs that are unlikely to provide a good conversion are filtered out. The basic input data required by the pipeline are the SNP 71mers (with the SNP at the 36^th^ position) and the reference genome. In this case, Bos taurus UMD3.1 was used as the reference genome.

Up to two independent probesets can be designed per SNP, one for each flanking sequence. The first step of the pipeline therefore consists in choosing which flank to use for the probe design. Each SNP flanking sequence is assigned to one of the following categories: “recommended”, “non-recommended” or “neutral”. The rules used to make the decision about the SNP flanking category are:

- The probe sequence must be unique in the target genome, therefore all the SNP sequences were aligned against UMD3.1 to confirm sequence uniqueness.
- The presence of interfering secondary polymorphisms within the first 20 nucleotides from the target SNP makes the sequence non-recommended. An interfering secondary polymorphism at a distance greater than 20 nucleotides makes the sequence neutral. In order to be recommended, no secondary polymorphism has to be present in the entire length of the flanking sequence.
- As an additional check, all the possible 16-mer generated from the flanking sequence were aligned against the reference genome and the total number of hits checked.
- At the core of the SNP sequence evaluation there is a machine learning algorithm that calculates the expected probability of conversion–p convert score–based on the SNP sequence composition. Only flanking sequences with a p convert score greater than 0.6 are recommended.

Once all the SNP sequences are evaluated, the pipeline adds to the array design the SNPs with at least one recommended flank. For such SNPs (in total 56960), only one probeset was created. For the SNPs with two recommended flanks, the sequence with the highest p-convert value was selected.

Additional 33,040 SNPs were selected to provide uniform predicted coverage of the buffalo genome and reach the total number of 90,000 SNPs, even though no recommended flank could be identified. In order to maximize the chance of conversion, for these additional SNPs two separate probesets (one for each flank) were designed.

The selected SNPnuon were aligned with the UMD3.1 bovine reference then queried against the buffalo reference sequence (NCBI GCF_000471725.1) to create a 71mer sequence flanking each SNP. Each SNP selected was named based on the (bovine) chromosome and position and the reference and alternative allele noted. In addition, if a SNP was present in the first 25 bases or last 25 bases of the 71mer, these were notated as 5' or 3' SNPs within the probe.

### GWAS

Blood samples were obtained for 619 Italian Mediterranean buffalo from 4 farms in the Lombardy region (Italy), and lactation records pedigree information was provided by the Italian Buffalo Breeders Association (ANASB). DNA was extracted from the blood sample as described above and genotyped with the Buffalo 90K Axiom Array.

Quality control (QC) on genotypes discarded replicated individuals and those with call rate (CR) lower than 10%. Only the best quality SNP probe category “PolyHighRes” called by the Affymetrix’ SNPolisher R package were retained and SNPs with a minor allele frequency (MAF) and a CR lower than 1% and 10% respectively were rejected. Preliminary analysis of the genetic structure of the population was conducted using the Multidimensional Scaling Plot statistics (Fig 2).

Associations between SNP genotypes and lactation records were tested by fitting all SNP simultaneously using the GRAMMAR procedure (Genome-Wide Rapid Association using Mixed Model and Regression), applied within the GenABEL package [38]. The GWA analysis was performed in a two-step procedure. First, lactation records were pre-corrected for fixed and polygenic effects which were included to account for genetic sub-structure, as higher or lower degree of genomic relationship between animals can have a direct impact on estimates, increasing false positives and negatives. The following model was used:

$$LactRecord_{ijkpqr}=\mu +Farm_{i}+CalvYear_{j}+CalvSeason_{k}+Calvings_{p}+Age_{q}+Polygenic_{r}+e_{ijkpqr}$$

where *LactRecord* is a 270 days in milk (DIM) conventional lactation record, *μ* is the general mean, *Farm* is a fixed farm effect (i = 1,4), *CalvYear* is a fixed effect for calving year (j = 1,2 for pre and post 2010), *CalvSeason* is a fixed effect for season of calving (k = 1,4), *Calvings* is a fixed effect for the number of calvings (p = 1,2 for primiparous and multiparous), *Age* is a covariate for age (in months), *Polygenic* is a polygenic effect for animal r, and *e* is the random residual, with e~ N(0,e^2^). The second step of the procedure involved the GWA study, using the random residual of each individual, obtained from the above model, as pre-corrected phenotype. A significance threshold, corrected for residual population inflation (lambda) but not for multiple testing, of *p* ≤ 1 x 10^−4^ was used to retain SNPs for further analyses.

### Pathway analysis

Putative genes 100kb down- and up-stream of all significant SNPs on at least one of the cross-validation runs were retrieved using Ensembl BIOMART [39], and used to obtain a network analysis of the results, using IPA^**®**^ Software (Ingenuity Pathway Analysis, www.ingenuity.com) and InnateDB for details on the genes identified by the network [40]. For IPA, the reference set was the Ingenuity Knowledge Base (genes only) and three analyses were conducted. The first to identify canonical pathways in which two or more genes were overrepresented and networks of genes were built. Finally, upstream regulators of focus genes were identified.
